# Physical Activity Reduces Clinical Symptoms and Restores Neuroplasticity in Major Depression

**DOI:** 10.3389/fpsyt.2021.660642

**Published:** 2021-06-09

**Authors:** Wanja Brüchle, Caroline Schwarzer, Christina Berns, Sebastian Scho, Jessica Schneefeld, Dirk Koester, Thomas Schack, Udo Schneider, Karin Rosenkranz

**Affiliations:** ^1^Faculty of Medicine, University Clinic of Psychiatry and Psychotherapie Luebbecke, Ruhr University Bochum, Bochum, Germany; ^2^Neurocognition and Action Group, Faculty of Psychology and Sports Sciences, Bielefeld University, Bielefeld, Germany; ^3^Department of Business Psychology, Faculty Business and Management, BSP Business School Berlin, Berlin, Germany

**Keywords:** neuroplasticity and exercise, major depression, paired associative stimulation, transcranial magnetic stimulation, physical activity

## Abstract

Major depressive disorder (MDD) is the most common mental disorder and deficits in neuroplasticity are discussed as one pathophysiological mechanism. Physical activity (PA) enhances neuroplasticity in healthy subjects and improves clinical symptoms of MDD. However, it is unclear whether this clinical effect of PA is due to restoring deficient neuroplasticity in MDD. We investigated the effect of a 3-week PA program applied on clinical symptoms, motor excitability and plasticity, and on cognition in patients with MDD (*N* = 23), in comparison to a control intervention (CI; *N* = 18). Before and after the interventions, the clinical symptom severity was tested using self- (BDI-II) and investigator- (HAMD-17) rated scales, transcranial magnetic stimulation (TMS) protocols were used to test motor excitability and paired-associative stimulation (PAS) to test long-term-potentiation (LTP)-like plasticity. Additionally, cognitive functions such as attention, working memory and executive functions were tested. After the interventions, the BDI-II and HAMD-17 decreased significantly in both groups, but the decrease in HAMD-17 was significantly stronger in the PA group. Cognition did not change notably in either group. Motor excitability did not differ between the groups and remained unchanged by either intervention. Baseline levels of LTP-like plasticity in the motor cortex were low in both groups (PA: 113.40 ± 2.55%; CI: 116.83 ± 3.70%) and increased significantly after PA (155.06 ± 10.48%) but not after CI (122.01 ± 4.1%). Higher baseline BDI-II scores were correlated with lower levels of neuroplasticity. Importantly, the more the BDI-II score decreased during the interventions, the stronger did neuroplasticity increase. The latter effect was particularly strong after PA (*r* = −0.835; *p* < 0.001). The level of neuroplasticity related specifically to the psychological/affective items, which are tested predominantly in the BDI-II. However, the significant clinical difference in the intervention effects was shown in the HAMD-17 which focuses more on somatic/neurovegetative items known to improve earlier in the course of MDD. In summary, PA improved symptoms of MDD and restored the deficient neuroplasticity. Importantly, both changes were strongly related on the individual patients' level, highlighting the key role of neuroplasticity in the pathophysiology and the clinical relevance of neuroplasticity-enhancing interventions for the treatment of MDD.

## Introduction

Major depressive disorder (MDD) is a common illness worldwide, with more than 264 million people affected (1). The pathophysiology of MDD is complex and likely due to different, possibly interacting mechanisms. Several preclinical and clinical studies described altered neuroplasticity in MDD (2–4), such as lower synaptic density in the brain which is associated with the severity of depressive symptoms (5).

Reduced LTP-like plasticity in the motor cortex has been described in MDD in studies using paired associative stimulation (PAS) as a specific transcranial magnetic stimulation (TMS) protocol (2, 6), which tests synaptic plasticity in the human brain (7). As this reduction showed some association with the symptom severity measured in clinical scales, developing interventions that aim at enhancing synaptic plasticity might be of crucial relevance in the treatment of MDD.

Physical activity (PA) is associated with higher levels of neuroplasticity in healthy subjects (8, 9), and has been identified as a protective factor against the onset of depression (10–13). The effect of PA or sports programs have been widely studied in MDD and the clinical benefit and therapeutic relevance has been shown (14–17) even of short-term interventions (18–22). Furthermore, PA seems to influence cognitive symptoms in MDD, such as deficits in attention, concentration, memory and executive functions (23, 24).

Most of these studies focused on measuring the effect of PA interventions using clinical outcome parameters, such as self- or investigator-rated depression scores, and did not investigate which neurobiological parameters, e.g., neuronal excitability or plasticity in the brain, might be associated with these clinical changes.

Our study investigated the effect of a PA program applied over a period of 3 weeks on clinical symptoms, neural excitability and PAS-induced plasticity in the motor cortex, as well as on cognitive performance in in-patients with an acute episode of MDD during their stay on the psychiatric ward. As most patients followed a more sedentary lifestyle before admission to hospital, we designed the PA program to be of moderate intensity, including elements of endurance, strength and coordination exercises that required interaction and teamwork of the participants, in order to avoid competition and the risk of perceived performance failure (25, 26). The effect of the PA program was compared to a control intervention (CI) administered over the same period of time that controlled for investigator-related effects, experience of group cohesion and social interaction, while the patients abstained from additional physical activity.

We expected—similar to previous studies—the level of PAS-induced plasticity to be low in MDD, and PA to lead to an improvement of clinical symptoms—at least of those known to be first indicators of symptom reduction, such as psychomotor retardation or loss of energy. Leading on from that, we asked (i) whether measures of PAS-induced plasticity and clinical symptoms are related on an individual subject's level, (ii) whether PA reverses neuroplasticity deficits in MDD, and (iii) whether PAS-induced plasticity could act as a marker to predict the clinical outcome of neuroplasticity enhancing interventions, such as PA.

## Materials and Methods

The study was approved by the Ethics committee of the Ruhr University Bochum, medical faculty in Bad Oeynhausen (Germany) and conducted in accordance with the Declaration of Helsinki.

### Subjects

After giving written informed consent, 50 in-patients meeting the clinical criteria of MDD as defined by the international classification of disease (ICD-10: F32, F33) were recruited (see Table 1 for details). The inclusion criteria were: (i) Age between 18 and 65 years, (ii) current depressive episode (BDI-II score ≥ 10 points; Hamilton depression score (HAMD-17) ≥ nine points), (iii) no concurrent brain stimulation treatment, (iv) no severe cardiovascular disease and body mass index <30 kg/m^2^, (v) no structural brain alteration as shown in brain imaging, and (vi) in case of concurrent medication: no major changes to antidepressant medication during the study; no medication with anticonvulsive medication or lithium; medication with benzodiazepine <1 mg/day lorazepam equivalent; no medication with antipsychotics in dosages known to alter brain excitability (27). Medication (e.g., with antidepressants) was continued as long as it complied with the inclusion criteria (see above) and left unchanged during the interventions.

**Table 1:** Patients' characteristics.

	**PA**	**CI**	***t*-test**
*N*	23	18	
Age (years ± SEM)	33.3 ± 3.06	40.11 ± 3.63	p=0.157
Age range (years)	18 – 63	18 – 65	
Sex	12 male: 11 female	11 male: 7 female	
Handedness	2 left: 21 right	1 left: 17 right	
Body mass index (kg/m^2^)	24.33 ± 0.93	25.69 ± 0.90	p=0.306
HAMD-17 (mean ± SEM) at M1	19.17 ± 0.78	17.83 ± 0.75	p=0.230
Severity (N)			
mild (9–16 points)	7	8	
moderate (17–24 points)	13	10	
severe (≥ 25 points)	2	0	
BDI-II (mean ± SEM) at M1	27.74 ± 1.44	26.11 ± 1.77	p=0.475
Severity (N)			
mild (10–19 points)	3	2	
moderate (20–29 points)	12	11	
severe (≥ 30 points)	8	5	

### Study Design

The study was performed within the setting of a primary care psychiatric university hospital on in-patients and ran for 18 months in total. Patients were screened according to the above mentioned criteria. In case of consent, they were recruited for participation in the study around 7–14 days after their admission to hospital, in order to allow for completion of diagnostics and for amelioration of severe symptoms of depression, such as suicidal ideation or agitation. They were informed that the purpose of the study was to compare the effect of two different interventions on their mood, their cognition and on neurophysiological parameters without implying a superior effect of one intervention to the other. As the recruitment rate of patients who fulfilled the inclusion criteria was expected to be low, the patients were successively recruited (rather than randomly assigned) in one of the two intervention groups and the investigators were not blinded. The first 25 patients were recruited into the PA group, and the succeeding 25 patients into the CI group. This approach ensured that 6–8 patients were participating at a time, which enabled interaction amongst participants during the sessions. Each patient participated for 3 weeks in one of the interventions. As neither the PA nor the CI sessions build up on each other, participants could join at any date.

**Figure 1:**
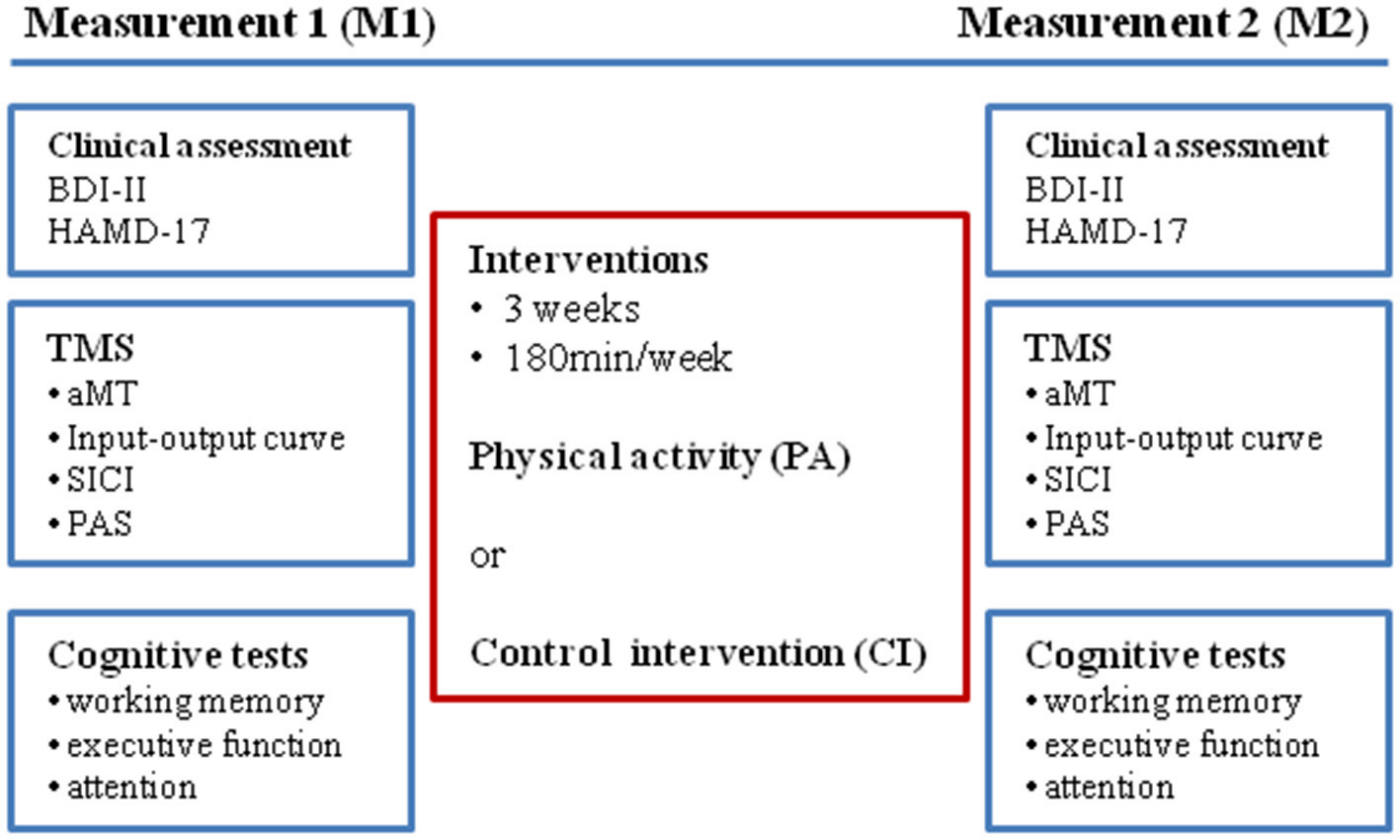
Experimental design.

Before the start (measurement 1; M1) and within 2–3 days after the end of the intervention period (measurement 2; M2) neurophysiological and cognitive parameters were tested (see Figure 1: experimental design), and clinical assessments by use of self- and investigator-related scales were performed.

### Interventions and Assessment of Physiological Parameters

Each intervention ran for 3 weeks. For each intervention the total duration was 180 min/week; thus the total intervention time was 540 min in 3 weeks.

The PA was performed on 3 days a week (Monday, Wednesday, Friday) and was guided by an instructor. Each session lasted 60 min (without breaks) and focused on one out of three exercise types once a week, either coordination, endurance or strength training. These three exercise sessions were repeated every week. The PA program aimed to increase motivation for and to induce a positive affective response towards physical and sportive activity. It mainly consisted of interactive games, which the patients had to perform within the group or with one patient as partner. This approach reduced competitiveness among participants which could trigger a sense of underachievement or failure, or could invoke negative prior experiences (e.g., with PA at school). In addition, each PA session started with a 10-min warm-up which combined physical and cognitive tasks, by coding certain movements (e.g., circling of arms, lifting knees up) with colors (colored cards held up by the instructor). During the warm-up participants walked briskly through the room and had to perform the required movement (once) when the instructor showed the respective colored card. The color-movement associations were changed randomly in every PA session.

The CI consisted of two sessions of 90 min each per week and was guided by an instructor. While the participants remained seated on chairs, they performed different games (logical puzzles, “black stories,” card games), which required them to interact and to cooperate with each other. In the logical puzzles pieces of information were given and required the participants to put them into a matrix and find out the missing pieces of information by deduction (e.g., *Who owned the zebra?*). The “black stories” were mysterious stories that required the participants to reconstruct what has happened by asking and guessing. The card games prompted the participants to cooperate and to exchange cards in order to play and win together against the rule of the game (e.g., *The Game—Spiel… solange Du kannst!*).

The CI controlled for the presence of and attention given by an instructor and the feeling of group cohesion. The participants were cognitively engaged (solving logical problems, memorizing facts, and forming strategies). Importantly, the participants of the CI group were not included in other PA programs of the psychiatric clinic, and were instructed not to engage in any physical or sportive activity beyond their routine activity (such as walking in the hospital and hospital garden). The clinical and nursing staff was informed about the study participation and monitored the participants activity accordingly.

During the PA and CI sessions the heart rate was measured using a pulse tracker fixed to the upper arm with an elastic band (OH1, Polar Electro Oy, Kempele, Finland). The mean heart rate at rest (before the start of the session) and during the session were calculated for each participant.

### Clinical Assessment

The Becks Depression Inventory II (BDI-II) was used for patients' self-assessment (28) and the Hamilton depression scale with 17 items (HAMD-17) was used for investigator-based assessment of clinical symptoms (29–31) once before and after the 3 weeks intervention period.

### Neurophysiology

#### Transcranial Magnetic Stimulation (TMS)

TMS was performed using a Magstim 200 stimulator connected to a figure-of-eight-shaped coil with an internal wing diameter of 70 mm (Magstim Company Ltd, Whitland, UK). At the start of the experimental sessions M1 and M2 the motor “hot spot” of the abductor pollicis brevis (ABP) muscle was determined. The coil was held with the handle pointing backwards and laterally 45° to the interhemispheric line to evoke anteriorly directed current in the brain and was optimally positioned to obtain motor evoked potentials (MEP) in the APB of the dominant hand (“hot spot”). The subjects were wearing a tight fitting cotton wool cap on which the coil position was marked using a soft tip pen in order to ensure that the coil was held in a constant position during the experimental session.

Stimulation intensities are quoted as percentage of maximal stimulator output (mean ± SEM).

#### EMG Recording

Surface electromyographic (EMG) recordings in a belly-to-tendon montage were made from the APB and the first dorsal interosseus (FDI) muscles of the dominant hand. The raw signal was amplified and filtered with a bandpass filter of 30 Hz to 1 kHz (Digitimer D360; Welwyn Garden City, UK). Signals were digitized at 2 kHz (CED Power1401, Cambridge Electronic Design, UK) and stored on a laboratory computer for offline analysis. Online EMG was used to control for muscle relaxation during data recording and trials showing voluntary muscle activation were discarded from the analysis (<1% of trials). The recordings of the FDI were only displayed on screen during the experiments in order to support the experimenter in holding the coil in a constant position and were not analyzed further.

#### Motor Excitability

At the start of each measurement (M1 and M2), the active motor threshold (aMT) and the stimulus intensity (SI) needed to evoke a MEP of ~1 mV peak-to-peak amplitude (SI_1mV_) were defined in the APB. To determine the aMT, the EMG pattern (rectified amplitude) under maximum voluntary contraction (MVC) was displayed on the screen and a marker line was set to determine 30% of this amplitude. The subjects were instructed to activate their APB (pressing the thumb down while their hand lies in a pronated position on a cushion placed on their lap) so that the EMG amplitude was as close to that marker line as possible. The TMS measurements were always performed by two experimenters. In the measurement of the aMT, one experimenter controlled the participants APB activation level to be constant and of defined strength. The aMT was defined as the minimum intensity needed to evoke a MEP of ≥200 μV in five out of 10 trials.

The SI_1mV_ was determined while the subjects were at rest with their hand muscles relaxed (as controlled by online EMG). Single TMS pulses were given (interstimulus interval 7 s) to determine the SI that gives a MEP of 1 mV peak-to-peak amplitude in five consecutive trials.

The input-output relationship of MEP amplitude to SI (IOcurve) was measured. For each SI of the IOcurve [50, 70, 80, 90, 100 (equal to SI_1mV_), 110, 120 130, and 150% of SI_1mV_] five consecutive TMS single pulses were applied with an interstimulus interval of 7 s and the MEPs recorded. The mean MEP amplitude per SI was calculated for each subject. Furthermore, the steepness of the IOcurve slopes defined as the steepness of the linear regression line through the given data points between 80 and 120% of SI_1mV_ (IOslope) were calculated.

#### Short Interval Intracortical Inhibition (SICI)

The short-interval intracortical inhibition [SICI curve; (32, 33)] was measured using subthreshold conditioning stimulus intensities of 70, 80, and 90% of active motor threshold (aMT) and two magnetic stimulators (MagStim 200) connected *via* a BiStim module (Magstim Company Ltd, Whitland, UK). The conditioning stimulus preceded the suprathreshold test stimulus (intensity set at SI_1mV_) by 3 ms (34).

Three blocks consisting of 30 trials each were performed. Each block examined one conditioning pulse intensity and consisted of 15 MEPs elicited by the test stimulus alone (test MEPs) and 15 conditioned MEPs presented in pseudorandom order (intertrial interval 7 s). The peak-to-peak amplitude of the conditioned and test MEPs was measured for each single trial to calculate the mean amplitude and percentage SICI (conditioned MEP/ test MEP; in %) for the three different conditioning stimulus intensities. This approach allowed us to measure the level of SICI at a single conditioning intensity as well as the recruitment of SICI (SICI curve) defined as the increase of SICI with increasing intensities of the conditioning stimulus.

#### Plasticity in the Motor Cortex as Assessed by Paired-Associative Stimulation (PAS)

PAS consisted of 200 electrical stimuli of the median nerve at the wrist of the relaxed dominant hand paired with a single TMS pulse (at SI_1mV_) over the contralateral hand motor cortex with a rate of 0.25 Hz. TMS single pulses were delivered through a figure-of-eight shaped coil (diameter of each wing 70 mm) connected to a Magstim 200 stimulator and was held in the same position as described above. Electrical stimulation (Digitimer DS7A) was applied through a bipolar electrode (cathode proximal), using square-wave pulses (duration 0.2 ms) at an intensity of three times the perceptual threshold.

The electrical stimuli preceded the TMS pulses by 25 ms (PAS25). PAS25 has been shown previously to induce a long-lasting MEP increase (7, 35, 36). Subjects were instructed to look at their stimulated hand and count the peripheral electrical stimuli they perceived; they were asked the actual count by the experimenter about three to four times during the application of PAS (37). During PAS, the MEPs evoked in the APB and FDI were displayed on-line on the computer screen to control for the correct coil position and stored for off-line analysis.

Before and 10 min after the end of the PAS-intervention 20 TMS pulses were delivered (intertrial interval 7 s) using SI_1mV_ and the MEPs recorded. Their mean amplitude was calculated. The effect of PAS was defined in each subject as change of the MEP amplitude in the APB (PASeffect = MEP after PAS / MEP before PAS; in %). In addition, PASchange refers to the change of PASeffects (PASchange = PASeffect after / before the intervention; in %).

#### Cognition

A PC-based test battery (Vienna test system, Schuhfried^®^, Austria) was used to measure different aspects of cognitive performance and executive function. Attention was tested using the work performance series which required subjects to perform additions and subtractions of single-digit numbers as fast and accurate as possible for 7 min. The Trail making test (part A and B) was used to assess the visuomotor processing speed and cognitive flexibility (38). The Response Inhibition (RI) task was used to assess voluntary control over responses within a changing context and required subjects to press a button as quickly as possible in reaction to a “Go” - signal (triangles) and to inhibit this reaction to an intermittently presented “NoGo” - signal (circle) (39, 40).

The Tower of London (TOL) assessed the planning abilities on the basis of clear rules and required the subjects to rearrange colored balls in a minimum number of moves (41, 42).

The STROOP interference test (color/word interference) was used to investigate the subjects' ability to control cognitive interference (43, 44).

Working memory performance was tested with the N-back verbal test [NBV; (45, 46)], a continuous performance measure (47, 48). A series of consonants was presented successively and subjects were required to press a button when the consonant displayed was identical to the one shown two places back (2-back paradigm) (49). To minimize the effect of familiarity, parallel versions of the tests were used in M2.

#### Data Analysis and Statistics

All data was tested for normal distribution by use of the Kolmogorov-Smirnov test. In case of not normally distributed data, non-parametric tests were used. All ANOVAs were tested for sphericity using Mauchly's test. In case of non-sphericity, Greenhouse-Geisser corrections were performed. Effect sizes (η^2^; *r*) were calculated for significant interactions. All data are given as mean ± SEM. Significance levels for the statistical tests are set to *p* ≤ 0.05; in case of multiple comparisons (results of cognitive tests) the significance level was adjusted to *p* ≤ 0.01.

The TMS parameters (aMT and SI_1mV_) of M1 and M2 were compared within groups by paired *t*-tests and between groups by unpaired *t*-tests. The IOcurve and SICI data were analyzed using ANOVA with the factor *group* (PA/CI) and the within-group factors *intervention* (before/after), *stimulus intensity* (IOcurve) or *conditioning pulse intensity* (SICI). The MEPs measured in M1 and M2 before PAS were compared by means of paired *t*-tests (within group) and unpaired *t*-tests (between groups) in order to control for correct adjustment of MEP size to 1 mV peak-to-peak amplitude. ANOVAs were performed on the raw data of MEPs with the factors *group, intervention* and *MEP amplitude before/after PAS*. For further analysis, the MEP raw data were normalized and expressed as percentage of MEPs (MEPs after PAS/ MEPs before PAS; PASeffect).

Correlations between neurophysiological data and clinical outcome (BDI-II and HAMD-17 scores) were calculated and significant results are reported giving Pearson's r for normally distributed and Kendall's tau for non-normally distributed data.

The raw data of cognitive tests was transformed into T-scores (mean = 50; SD = 10); with T-scores >50 indicating higher, and T-scores <50 indicating lower performance in comparison to a representative population (matched for age, sex and level of education) as given by the Vienna Test System (Schufried^®^, Austria). Similar to the analysis of the neurophysiological data, ANOVAs were performed with the between group factor *group* and the factors *intervention* as within-group factor. *Post-hoc* tests were performed when necessary and the significance level was adjusted to correct for multiple comparisons (see above).

## Results

### Subjects' and Clinical Data

Out of 50 recruited patients, 23 (of 25) in the PA group and 18 (of 25) in the CI group finished the study. Two patients of the PA group and three patients of the CI group were discharged from hospital before the M2 measurements could be taken. In addition, in the CI group two patients required emergency treatment and two patients discontinued their participation. Only results of patients who participated in both measurements (M1 and M2) and in all intervention sessions during the 3 weeks intervention period are reported. The patients in the PA and CI groups were comparable with regard to age, body mass index, BDI-II and HAMD-17 scores at M1 (see Table 1 for details).

### Clinical Assessment

**Figure 2:**
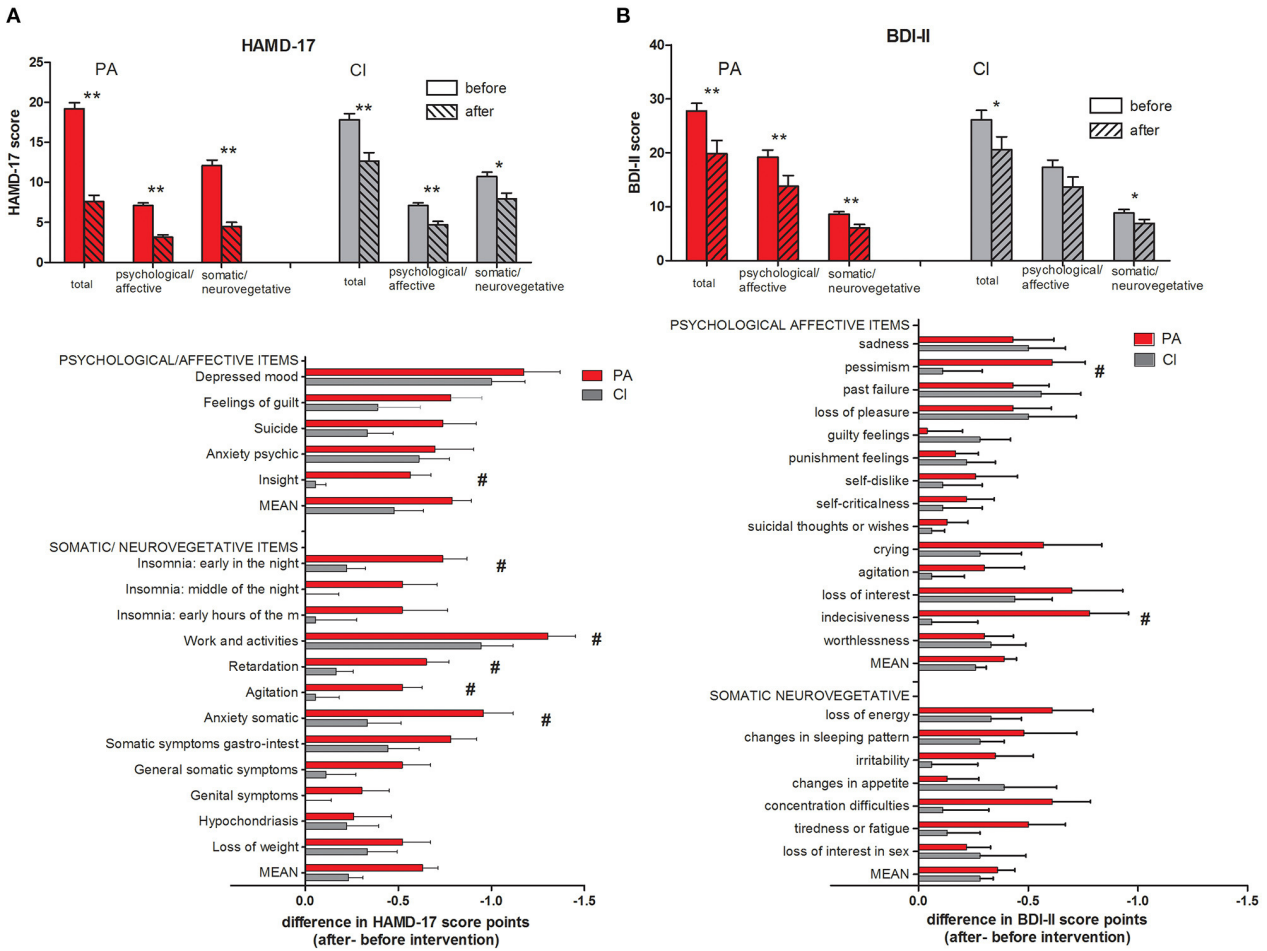
HAMD-17 **(A)** and BDI-II **(B)** scores with subscales. The mean scores of all items (total) as well as subscores for psychological/affective and somatic/neurovegetative items are given (mean ± SEM; *t*-test: **p < 0.001; *p < 0.01). Below the score change (after – before intervention) is given for each single item of the questionnaire (*t*-test; ^#^*p* < 0.05).

The subjects in the PA and CI groups did not differ in their BDI-II and HAMD-17 scores before the start of the intervention (unpaired *t*-tests, n.s.; see Table 1 and Figure 2). After the intervention, the BDI-II and HAMD-17 scores decreased significantly in both groups (paired *t*-tests; *p* < 0.009; see Figure 2) while the decrease in HAMD-17 score (HAMD-17 after—before intervention) was significantly stronger in the PA group (unpaired *t*-test; *p* < 0.001), there was no between-group difference in the decrease of the BDI-II score. A two-way ANOVA with the factors *group* (PA/CI) and *intervention* (before/after) showed for the HAMD-17 scores a significant interaction [ANOVA; *F*_(1,39)_ = 16.52; *p* < 0.001], and significant main effects of the factor *group* [ANOVA; *F*_(1,39)_ = 4.41; *p* = 0.042] and the factor *intervention* [ANOVA; *F*_(1,39)_ = 112.94; *p* < 0.001]. For the BDI-II score data, there was no significant interaction, but a main effect of *intervention* [ANOVA; *F*_(1,39)_ = 27.19; *p* < 0.001].

A detailed analysis of the single items of the HAMD-17 scores (see Figure 2A) showed that the decrease in the items “insight,” “insomnia: early in the night,” “work and activities,” “retardation,” “agitation,” and “anxiety somatic” was significantly stronger in the PA group than in the CI group. For the BDI-II scores (see Figure 2B), the score for the items “pessimism” and “indecisiveness” decreased significantly stronger in the PA group than in the CI group.

The BDI-II and HAMD-17 scores measured before and after the interventions did not show a significant correlation in either the PA or the CI group.

### Physiological Parameters

Before and during the interventions, the subjects' heart rate was continuously monitored. At rest, the mean heart rates were not different in the PA [69.00 ± 2.03 beats per minute (bpm)] and the CI group (71.39 ± 3.19 bpm; *t*-test: n.s.). During PA, the mean heart rate increased to 126.84 ± 2.83 bpm, showing that the patients were exercising with moderate intensity. In the CI group, the heart rate increased slightly to 83.02 ± 3.08, but this increase was not significantly different from their baseline mean heart rate (paired *t*-test; n.s.). The mean heart rates were not correlated to any neurophysiological or clinical parameters in either group.

### Motor Cortical Excitability and Short-Interval Intracortical Inhibition

**Figure 3:**
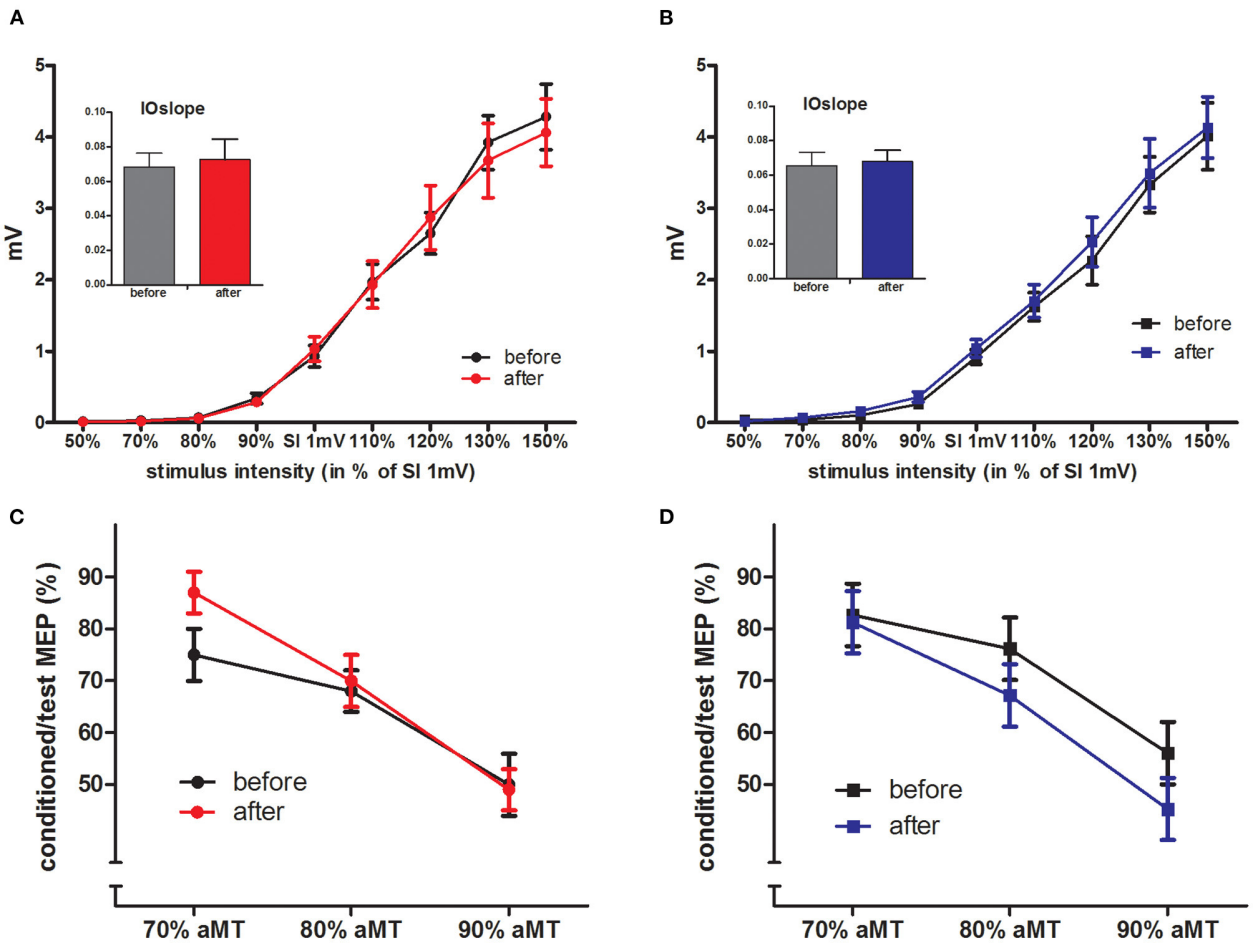
IOcurves and SICI. **(A,B)** displays the IOcurves measured in PA **(A)** and CI **(B)** groups before and after the intervention, with the IOslope given in the inserted diagram (mean ± SEM). IOcurves and IOslopes were not changed by the interventions within the groups, and there were no between-group differences. **(C,D)** displays the SICI results for PA **(C)** and CI **(D)** groups, again there were no differences within the groups (before/after intervention) nor between the groups.

The aMT and SI_1mV_ were not different between the two groups (see Table 2). The IOcurves (see Figures 3A,B) showed an increase of MEP amplitudes with increasing TMS intensity. The IOcurves measured before and after the interventions were not different in either the PA or the CI group (ANOVA, main effect of *stimulus intensity p* < 0.001; no effect of *intervention*). Furthermore, there were no group differences between the IOcurves (ANOVA; main effect *group*: *p* = 0.96; no significant interaction). The SICI (see Figures 3C,D) was stronger with increasing conditioning stimulus intensities. Again, there was no difference in SICI before and after the intervention within each group (ANOVAs; main effect of *conditioning stimulus intensity*: *p* < 0.001; effect of *intervention*: n.s.), nor was there any difference between the groups (ANOVA, main effect of factor *group*: *p* = 0.775; no significant interactions).

**Table 2:** TMS and PAS parameters and MEP amplitudes.

	**PA**		**CI**	
**TMS parameters (% stimulator output)**	**Mean**	**SEM**	**Mean**	**SEM**
aMT M1	34.96	± 1.22	33.17	± 1.09
aMT M2	34.78	± 0.84	35.50	± 1.12
SI_1mV_ M1	51.87	± 1.75	51.89	± 2.12
SI_1mV_ M2	51.74	± 1.59	53.89	± 1.79
**PAS parameters**				
Sensory threshold M1 (mA)	0.28	± 0.02	0.36	± 0.02
Sensory threshold M2 (mA)	0.27	± 0.06	0.36	± 0.02
Sensory stimuli counted M1 (N)	201.05	± 0.40	199.06	± 0.76
Sensory stimuli counted M2 (N)	200.52	± 0.63	199.22	± 0.68
**MEP amplitudes (mV)**				
IOcurve MEP (SI_1mV_) M1	0.93	± 0.15	0.92	± 0.09
IOcurve MEP (SI_1mV_) M2	1.03	± 0.17	1.04	± 0.12
SICI test MEP M1	1.01	± 0.14	1.02	± 0.15
SICI test MEP M2	0.99	± 0.15	0.99	± 0.13
MEP before PAS M1	1.15	± 0.15	0.93	± 0.09
MEP after PAS M1	1.29	± 0.16	1.09	± 0.11
MEP before PAS M2	0.99	± 0.10	1.08	± 0.12
MEP after PAS M2	1.45	± 0.16	1.34	± 0.17

### Motor Cortical Plasticity - PAS

**Figure 4:**
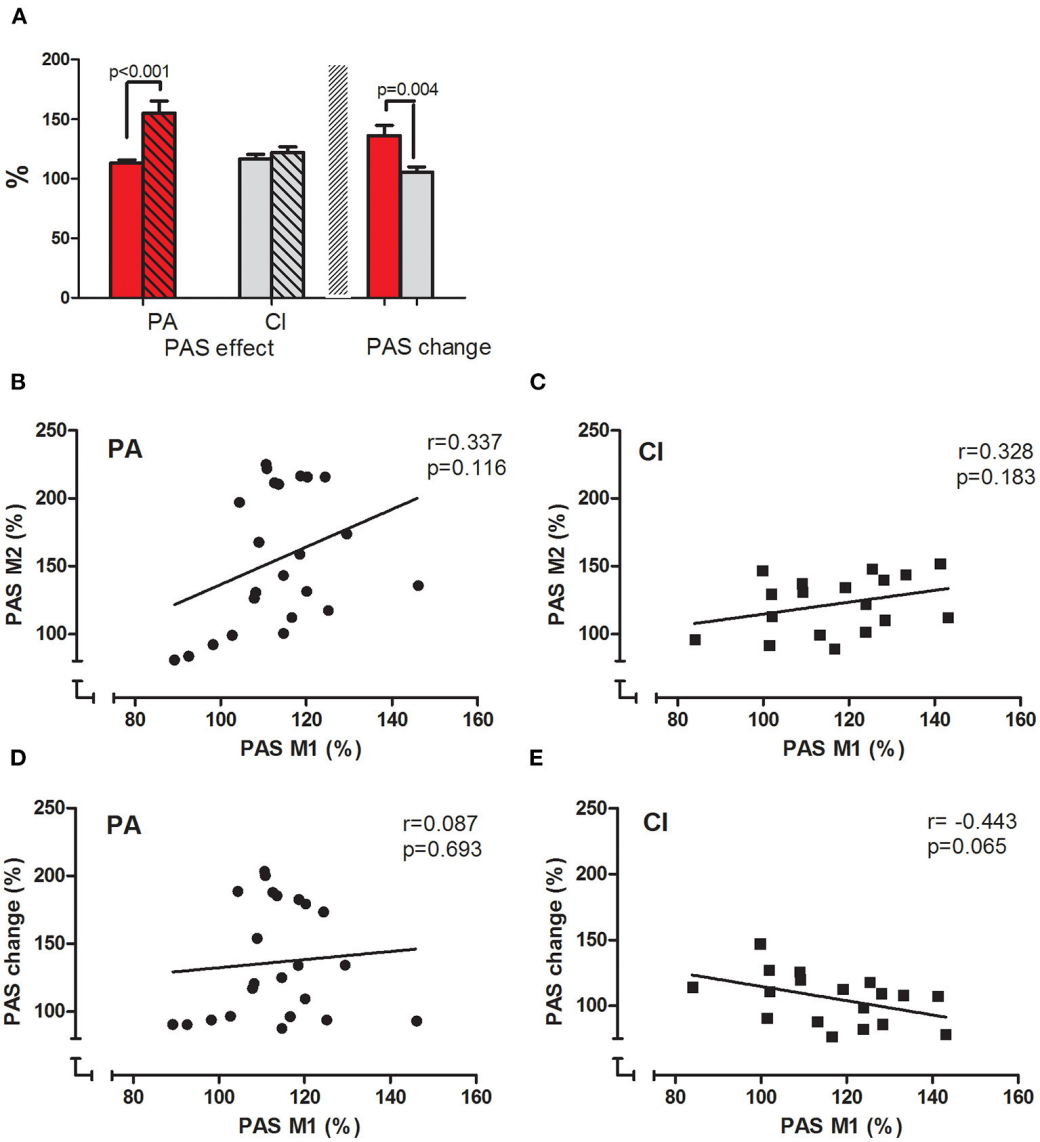
PASeffect and correlations. **(A)** Displays the PASeffects measured before (clear bars) and after (hatched bars) the interventions in PA (red bars) and CI (gray bars) groups. The PASeffect increased significantly after the intervention in the PA group, but not in the CI group (*t*-test results are given). **(B,C)** display the correlation between the PASeffects measured before and after the intervention in PA **(B)** and CI **(C)** groups; **(D,E)** display the correlation between the PASeffect measured before the intervention and PASchange for each group [PA: **(D)** and CI: **(E)**]. The results of Pearson's correlation are given.

The mean MEP amplitudes measured before PAS were not different within each group when tested either before or after the intervention (paired *t*-tests, n.s.), and not different between the groups (unpaired *t*-tests: n.s.); thus ensuring comparability between the groups. In the PA group, PAS induced a significant increase (paired *t*-tests; *p* < 0.001) of the mean MEP amplitude before and after the intervention. The PASeffect (MEP after/before PAS, in %) was significantly stronger after PA (before: 113.40 ± 2.55%; after: 155.06 ± 10.48; paired *t*-test; *p* < 0.001; see Figure 4A).

In the CI group, PAS also significantly increased the mean MEP amplitude before and after the intervention (paired *t*-tests; *p* < 0.001). However, there was no difference in the PASeffects measured before and after the CI (before: 116.83 ± 3.70%; after: 122.01 ± 4.91%; paired *t*-test; n.s.; see Figure 4A).

The interaction of the factor *intervention* (before/after) and *group* (PA/CI) was significant [ANOVA; *F*_(1, 39)_ = 9.09; *p* = 0.005; η^2^ = 0.40; *r* = 0.63], as were the main effects of *intervention* [*F*_(1, 39)_ = 14.98; *p* < 0.001] and *group* [ANOVA; *F*_(1, 39)_ = 4.15; *p* = 0.048]. The PASchange was significantly higher in the PA than in the CI group (unpaired *t*-test; *p* = 0.004).

The PASeffects measured before and after the intervention were only weakly correlated in each group (PA: Pearson's *r* = 0.337, *p* = 0.116; CI: Pearson's *r* = 0.328, *p* = 0.183; see Figures 4B,C). While the PASeffect at baseline and the PASchange showed no correlation in the PA group (Pearons's *r* = 0.087, *p* =0.693, see Figure 4D), there was a weak but non-significant correlation in the control group (Pearson's *r* = −0.443, *p* = 0.065; see Figure 4E). Thus, the baseline PASeffect did neither predict the PASeffect after the intervention, nor PASchange.

### Correlations Between Clinical Scales and PAS

**Figure 5:**
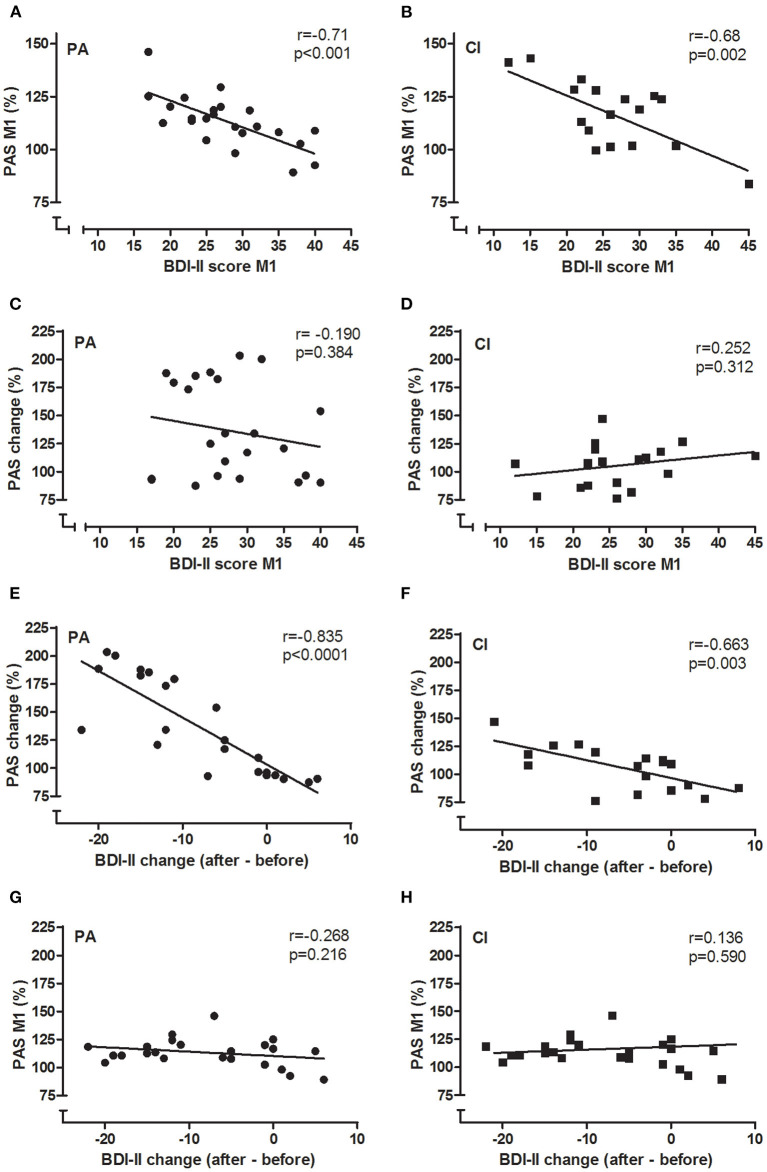
Correlations between BDI-II and PAS. The correlations of BDI-II and PAS are given for PA **(A,C,E,G)** and CI **(B,D,F,H)** groups. Pearson's *r* and *p*-values are given in each figure. BDI-II and PASeffect measured before the intervention (M1) were negatively correlated in each group **(A,B)**: the higher the BDI-II score, the smaller was the PASeffect. Similarly, the change of BDI-II and PASeffects by the intervention were negatively correlated in each group **(E,F)**. The BDI-II at baseline did not predict the amount of PASchange that could be induced by the interventions **(C,D)**, neither did the PASeffect at baseline (M1) predict the amount of BDI-II change **(G,H)**.

In both groups, there was a significant negative correlation between the BDI-II scores and the PASeffect measured before the interventions (see Figures 5A,B; PA: Pearson's *r* = −0.71, *p* < 0.001; CI: Pearson's *r* = −0.68, *p* = 0.002; for both groups together: Pearson's *r* = −0.695, *p* < 0.001): with increasing BDI-II scores the PASeffect was less strong. Furthermore, the change of the BDI-II scores and the change of the PASeffect by the intervention were significantly correlated in each groups (see Figures 5E,F; PA: Pearson's *r* = −0.835; *p* < 0.001; CI: −0.663, *p* = 0.003): the stronger the BDI-II score decreased, the stronger did the PASeffect increase. The latter effect was more prominent in the PA group (see Figure 5E).

The BDI-II scores at baseline were not correlated to the PASchange in either of the groups (Figures 5C,D; PA: *r* = −0.19; *p* = 0.384; CI: *r* = 0.252; *p* = 0.312); thus, the baseline BDI-II did not predict the PASchange. Furthermore, the change of BDI-II scores were not correlated to the PASeffect at M1 in either group (Figures 5G,H; PA: *r* = −0.268; *p* = 0.216; CI: *r* = 0.136; *p* = 0.59), showing that the PASeffect at baseline did not predict the clinical effect of the interventions as shown in reduction of BDI-II scores.

There were no correlations between the HAMD-17 scores and the PAS data in either group; and no correlations between clinical scales and other neurophysiological parameters.

### Cognition

Table 3 shows the results of the cognitive test performed before and after the intervention in each group (mean T-scores ± SEM). After the intervention, there was a significant increase of T-scores of some parameters within groups that indicated an improved performance (see results of paired *t*-tests in Table 3). There were no between-group differences in cognitive test before or after intervention, apart from a significant difference in the STROOP test (Δ after-before: baseline reading), which is of minor relevance in the absence of differences in the interference conditions.

**Table 3:** Results of cognitive tests (T-scores).

	**PA**					**CI**				
**Attention and working speed**		**Mean**		**SEM**	**Paired *t*-test**		**Mean**		**SEM**	**Paired *t*-test**
**Work performance series**										
Numbers worked	Before	48.96	±	1.73		Before	52.89	±	3.03	
	After	53.35	±	1.72	*p* < 0.001	After	54.78	±	2.43	n.s.
	Δafter-before	4.39	±	0.59		Δafter-before	1.89	±	1.63	
Errors	Before	51.83	±	2.22		Before	42.89	±	3.03	
	After	52.26	±	2.21	n.s.	After	50.00	±	1.57	*p* < 0.05
	Δafter-before	0.43	±	1.99		Δafter-before	7.11	±	3.00	
**Trail making test**										
Part A	Before	49.61	±	0.95		Before	51.28	±	1.54	
	After	54.39	±	1.41	*p* < 0.001	After	54.78	±	2.50	n.s.
	Δafter-before	4.78	±	1.08		Δafter-before	3.47	±	1.79	
Part B	Before	52.83	±	1.27		Before	50.39	±	2.13	
	After	58.87	±	1.65	*p* < 0.001	After	54.83	±	2.31	*p* < 0.001
	Δafter-before	6.04	±	1.27		Δafter-before	4.44	±	1.03	
**Executive function**		**Mean**		**SEM**	**Paired** ***t*****-test**		**Mean**		**SEM**	**Paired** ***t*****-test**
**STROOP**										
Baseline reading	Before	52.13	±	1.54		Before	51.50	±	2.02	
	After	57.57	±	1.74	*p* < 0.001	After	52.50	±	2.11	n.s.
	Δafter-before	5.43	±	0.93		Δafter-before	1.00	±	1.13	
Baseline naming	Before	52.00	±	1.79		Before	50.28	±	2.34	
	After	55.57	±	1.91	*p* < 0.001	After	51.83	±	1.95	n.s.
	Δafter-before	3.57	±	0.88		Δafter-before	1.56	±	1.91	
Interference reading	Before	49.96	±	1.51		Before	45.50	±	2.96	
	After	50.48	±	1.80	n.s.	After	46.44	±	2.02	n.s.
	Δafter-before	0.52	±	1.94		Δafter-before	0.94	±	2.64	
Interference naming	Before	51.48	±	2.06		Before	52.89	±	2.67	
	After	50.22	±	1.80	n.s.	After	48.33	±	1.65	n.s.
	Δafter-before	−1.26	±	1.77		Δafter-before	−4.56	±	2.73	
**Response Inhibition**										
Commission errors	Before	48.61	±	1.96		Before	49.06	±	2.60	
	After	52.26	±	2.17	*p* < 0.05	After	51.33	±	2.76	n.s.
	Δafter-before	3.65	±	1.40		Δafter-before	2.28	±	2.16	
Omission errors	Before	44.78	±	1.97		Before	45.89	±	2.19	
	After	49.39	±	1.82	*p* < 0.05	After	48.72	±	2.25	n.s.
	Δafter-before	4.61	±	1.80		Δafter-before	2.83	±	1.96	
Sensitivity index	Before	46.74	±	2.25		Before	47.78	±	3.01	
	After	52.13	±	2.20	*p* < 0.01	After	50.28	±	2.60	n.s.
	Δafter-before	5.39	±	1.57		Δafter-before	2.50	±	2.09	
**Tower of London**										
	Before	55.83	±	2.00		before	54.22	±	1.61	
	After	57.87	±	1.35	n.s.	after	55.56	±	2.51	n.s.
	Δafter-before	2.04	±	1.85		Δafter-before	1.33	±	2.59	
**Working memory**	**Mean**	**SEM**	**Paired** ***t*****-test**		**Mean**		**SEM**	**Paired** ***t*****-test**		
**N-back-verbal**										
Correct answers	Before	55.65	±	3.94		Before	54.61	±	3.95	
	After	73.22	±	2.76	*p* < 0.001	After	67.00	±	4.03	*p* < 0.01
	Δafter-before	17.57	±	4.49		Δafter-before	12.39	±	3.76	

Although there were no significant differences on how the performance was influenced by the intervention between the two groups, T-scores tended to increase stronger in the PA group: here the stronger increase of T-scores in Trail making and N-back tasks might indicate a stronger improvement of attention/ working speed and of working memory, respectively.

## Discussion

Our study investigated the effect of PA—in comparison to a CI—on neuronal excitability and plasticity, as well as on clinical and cognitive symptoms in MDD. Confirming previous studies, we showed that (i) PA had a beneficial clinical effect as such as it reduced the severity of symptoms, such as psychomotor retardation and loss of energy as assessed by HAMD-17 and known to improve early in the course of MDD; and that (ii) the baseline level of motor cortical LTP-like plasticity is low in MDD.

Our study now crucially expands these findings by showing, that (iii) the severity of psychological/affective symptoms of MDD, as monitored with the BDI-II is highly correlated to the amount of LTP-like plasticity, and (iv) that PA as intervention can normalize deficient neuroplasticity which—in turn—is correlated to the reduction of clinical symptoms. In addition, (v) working memory performance (N-back verbal test), executive functions (Response Inhibition) and cognitive working speed (Trail making test) tended to improve stronger after PA than after the CI.

The reduction of PAS-induced LTP-like plasticity in the motor cortex in MDD before the interventions confirms findings of previous studies (2, 6), and different mechanisms may account for this. First, changes in structural and functional synaptic plasticity, such as reduced synaptic density in dorsolateral prefrontal cortex (DLPFC), the anterior corpus callosum (ACC), and the hippocampus (5, 50, 51) are described in MDD, likely leading to reduced functional connectivity within and between networks underlying mood and cognition. The motor cortex is an important node in the brain and processes information from various inputs (52, 53) as it is strongly interconnected with numerous brain areas. Several key structures of the cognitive network, such as the DLPFC (54), the posterior parietal cortex (55), as well as frontal areas (56) have been shown to be connected to the motor cortex, as several double-pulse TMS studies (57–60) as well as cortico-cortical paired associative stimulation studies have shown (61–64). Therefore, the PASeffect measured in the motor cortex represents a valid surrogate marker for plasticity in the networks that play a key role in the pathophysiology of MDD.

Second, the induction of LTP-like plasticity depends on postsynaptic activation of NMDA-receptors (35), and the alterations in the glutamatergic system described in MDD (65) are likely to contribute to a reduction of PASeffects.

Lastly, the presence of hallmark symptoms of MDD, such as anhedonia, loss of interest and of motivation, and psychomotor retardation, might further contribute to a reduction of synaptic plasticity. A lack of physical (66) and cognitive activity, and of social interaction, deprives the brain of important stimuli, which consequently might contribute to the downscaling or loss of synapses, which are necessary to keep the brain susceptible to plastic changes (67). Enhancing neuroplasticity is therefore a promising treatment approach, and using PA as an intervention has been proven to be clinically beneficial in MDD.

There is good evidence that PA modifies structural and functional brain plasticity (68–70). PAS-induced plasticity is higher in physically active healthy subjects compared to those with a sedentary lifestyle (8, 71). PA has been shown to increase metabolism and oxygenation, to modulate neurotransmitters and the release of neurochemical and neurotropic factors in the brain (72–77), and by these mechanisms likely contributes to the enhancement of plasticity.

After the 3 weeks of intervention, the PASeffect increased stronger in patients of the PA than of the CI group. Since parameters of neural excitability, the IOcurve and SICI, did not change, this is likely to be due to enhanced LTP-like plasticity after PA, rather than stronger motorneuronal recruitment or a reduction of GABAergic inhibition. Importantly, the PASeffect measured before the interventions was not related to PASchange, which precludes a saturation effect (“ceiling effect”).

We monitored the intensity of the PA program by measuring the heart rate, and its rise to moderate levels during the PA session indicated a moderate level of physical strain for the patients; while there was no notable change of heart rate in patients of the CI group. Given the moderate intensity and short duration of the PA program, the strength of its effect on PAS-induced plasticity in MDD was surprising. In the context of synaptic density and activity being reduced in MDD (5), it might point toward enhanced susceptibility to undergo LTP-like plasticity induction, in terms of a homeostatic mechanism. Similar to increased plasticity after sensory deprivation (78), the PA program might have re-activated synaptic connections and consequently “boosted” the efficiency of the PAS protocol to induce LTP-like plasticity (79).

Previous studies using PAS (6) and measures of synaptic density (5) have described an association between the severity of depressive symptoms and neuroplasticity in MDD. We extended these findings in our study by showing that the amount of LTP-like plasticity strongly correlates with the BDI-II scores in both groups at baseline; and further, that the amount of BDI-II score reduction and the increase of PAS plasticity seen after the interventions were correlated in both groups, though stronger in the PA. However, as the baseline PASeffect was not correlated to the BDI-II score change, and the baseline BDI-II was not correlated to the PASchange, the value of the PASeffect or the BDI-II score measured before the interventions is limited with regard to predicting either the clinical outcome or the amount of neuroplastic change.

There were no such correlations of the HAMD-17 and PASeffects, nor of the HAMD-17 and BDI-II scores. However, the difference in clinical outcome was shown in the HAMD-17, which decreased significantly stronger in the PA than in the CI group.

Several studies in MDD have already shown that self- and observer-rated scales are only moderately correlated, if at all (80, 81). Compared to the BDI-II (82), the HAMD-17 (83) has a higher sensitivity to depict changes (81, 84). The scores are quite different with regard to their item structure. The BDI-II focuses more on psychological/affective and the HAMD-17 more on somatic/neurovegetative items. As a self-rated score, the BDI is dependent on the patients' self-perception which—in turn—is often compromised by the symptoms of depression themselves. As it is focussing more on cognitive symptoms, the BDI-II is likely to be less sensitive to change because cognitive symptoms are more persistent than somatic symptoms in the course of treatment (85–87). Thus, the BDI-II has probably not been sensitive enough to depict early symptom changes that might have evolved during the 3 weeks observational period. The HAMD-17 is investigator-rated, focuses stronger on somatic/neurovegetative symptoms that are more likely to remit at an earlier phase in the course of treatment, and therefore is more perceptive to change over shorter observation periods (81). Furthermore, an observer could be more likely to see improvement in depressive symptoms than a patient affected by a cognitive bias (86, 87).

Thus, if there are first signs of symptom remittance, they are more likely to present as a reduction in HAMD-17 than in BDI-II; as was the case for the patients of the PA group. The correlation of the BDI-II and PAS-induced neuroplasticity might indicate, that factors like loss of interest, indecisiveness and pessimism, which were most strongly reduced in the PA group, might be more closely associated to deficient neuroplasticity and therefore sensitive to plasticity enhancing interventions.

The patients' attention, working memory and executive functions as tested by the cognitive test battery were not notably different from the age- and education-matched healthy control group (implemented in the test battery). Similar to other studies our findings might indicate that patients with MDD achieve this level of performance by a compensatory higher level of brain network activation, and thus their cognitive capacity is compromised by recruiting more brain resources as healthy controls (88).

Though there were no statistically significant differences, the cognitive performance tended to increase more in the PA than CI groups after the interventions, hinting toward an increased cognitive capacity by PA, as similarly described in physically active healthy subjects (89–91).

The study was performed on patients during their stay on the psychiatric ward and thus the duration of the interventions was limited to 3 weeks which might have been too short a period to induce differentiated effects on cognitive symptoms. Furthermore, we tested neuroplasticity, clinical and cognitive symptoms directly after the end of the interventions. Future studies need to address how long the PA-induced changes might last for and how they might be used to facilitate standard treatment of MDD.

In summary, we showed that a PA intervention supports the remission of clinical symptoms and normalizes deficient LTP-induced neuroplasticity in MDD, and that these two observations are highly correlated. Our study therefore further highlights the role of neuroplasticity in the pathophysiology of MDD and of PA in its treatment by showing that this intervention directly targets the deficient neuroplasticity as an underlying pathophysiological mechanism. Further research is needed to explore whether the effect of therapeutic interventions, might be predicted by clinical or neurophysiological parameters, as this would support the development of individualized treatments strategies.

## Data Availability Statement

The original contributions presented in the study are included in the article/supplementary material, further inquiries can be directed to the corresponding author/s.

## Ethics Statement

The studies involving human participants were reviewed and approved by Ethics committee of the Ruhr University Bochum in Bad Oeynhausen/ Germany. The patients/participants provided their written informed consent to participate in this study.

## Author Contributions

WB, DK, and KR contributed to the experimental design. WB, CS, CB, SS, JS, and KR performed the experiments and contributed to the data acquisition. WB and KR analyzed and interpreted the data and wrote the manuscript. DK, CS, TS, and US contributed to the interpretation of the results. All authors gave their final approval of the version to be published.

## Conflict of Interest

The authors declare that the research was conducted in the absence of any commercial or financial relationships that could be construed as a potential conflict of interest.
